# Supplementary material for: Foxo3 Knockdown Mediates Decline of Myod1 and Myog Reducing Myoblast Conversion to Myotubes
Source: Cells. 2023 Aug 29;12(17):2167. doi: 10.3390/cells12172167 (PMC10486649; doi:10.3390/cells12172167)
Supplement: Supplementary file 1 [file cells-12-02167-s001.zip › cells-2559191-supplementary material.pdf]

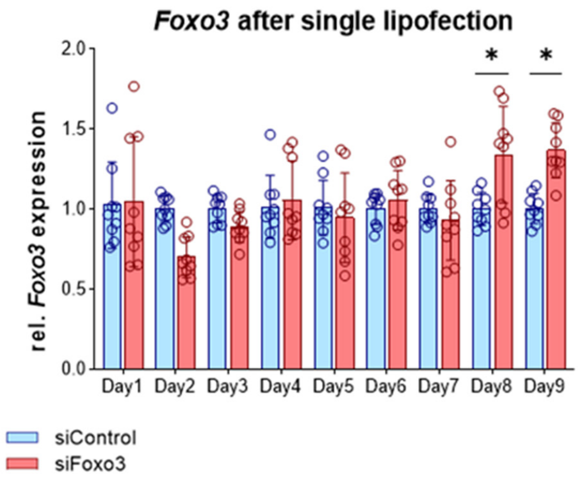

Figure S1. Lipofectamine mediated siRNA does not show a *Foxo3* knockdown on transcriptional level (A) During differentiation, there is no significant *Foxo3* knockdown detectable on the transcriptional level (by Lipofectamine) - mediated siRNA RNAi. A significant *Foxo3* overexpression was observed on day 8 and day 9 ($n = 9$, $p < 0.05$). Mean ± SD. Significant differences are depicted as *($p < 0.05$).

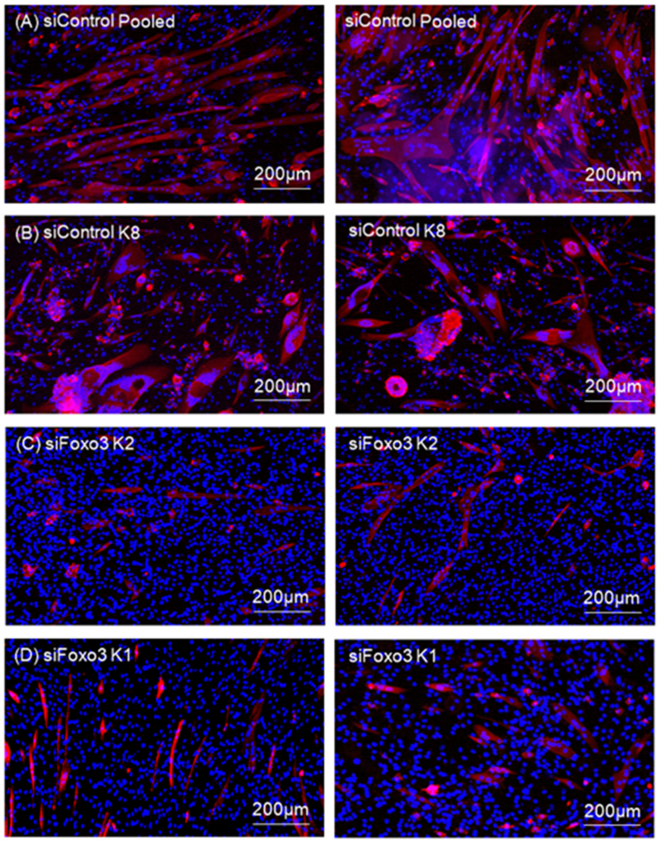

Figure S2. Long-term Foxo3 knockdown leads to smaller myotubes and a lower number of nuclei per myotube (A-B) Immunofluorescence staining for MHC of siControl pooled and siControl K8. Representative images indicate bigger myotubes with a high number of nuclei inside the myotubes. (C-D) Immunofluorescence staining for MHC of siFoxo3 K2 and siFoxo3 K1.

Representative images show smaller myotubes and a lower number of nuclei per myotube due to *Foxo3* knockdown.
